# Outcomes of Cardiac Repair in Heterotaxy Syndrome Patients—Tertiary Center Experience

**DOI:** 10.1016/j.cjcpc.2025.06.005

**Published:** 2025-07-14

**Authors:** Mohammad A. Ebrahim, Nada T. Alzayed, Sakeena M. Alsahhaf, Mohammad A. Almulla, Karim M. Yassin, Leina A. Shabaan, Moustafa A. Elsayed, Vadim G. Lyubomudrov

**Affiliations:** a Department of Pediatrics, Kuwait University Faculty of Medicine, Affiliated with Chest Diseases Hospital, Jabriya, Kuwait; b Primary Healthcare, Ministry of Health, Al Sabah Health Area, Kuwait; c Department of Medicine, Ministry of Health, Al Sabah Health Area, Kuwait; d Department of Medicine, Ministry of Health, Al Sabah Health Area, Kuwait; e Department of Medicine, Ministry of Health, Al Sabah Health Area, Kuwait; f Department of Cellular, Anatomical and Physiological Sciences, University of British Columbia, Vancouver, British Columbia, Canada; g Department of Pediatric Cardiac Surgery, Chest Diseases Hospital, Ministry of Health, Al Sabah Health Area, Kuwait

**Keywords:** arrhythmia, heterotaxy syndrome, surgical outcomes

## Abstract

**Background:**

This study aims to determine factors associated with poor outcomes and frequency of arrhythmia incidence in patients with heterotaxy syndrome (HS).

**Methods:**

A retrospective cohort study in a single tertiary center was conducted and included patients with operated HS between 2011 and 2020. A total of 52 patients were included. Relevant factors associated with mortality that were considered included univentricular (UV) or biventricular (BV) physiology, prematurity, low weight/age at surgery, the presence of atrioventricular valve regurgitation, anomalous pulmonary veins drainage, and type of atrial isomerism.

**Results:**

Thirty-three patients (63.4%) were diagnosed with left atrial isomerism (LAI), whereas the remaining 19 (36.5%) were diagnosed with right atrial isomerism (RAI). Thirty-eight patients (73%) underwent UV repair, whereas 14 patients (27%) had BV physiology. Patients were followed up for an average of 4.8 years. Lastly, 14 patients (27%) had died during the follow-up period. Notably, most patients with complete repair are among the LAI group, and high-grade heart block only occurred in patients with LAI. Moreover, patients with LAI were more likely to receive BV repair, whereas patients with RAI were more likely to undergo UV repair (*P* = 0.008). In addition, pulmonary venous anomalies occurred more frequently among the RAI group. Expectedly, nonsinus rhythm was frequently present among the cohort. Finally, the mortality rate was significantly higher among the RAI group (42% vs 18%, *P* = 0.06).

**Conclusion:**

Managing HS patients with UV physiology still remains a challenge. Risk factors for mortality included lower initial operation age, and RAI approached significance. These data may assist with risk stratification and patient counseling.

## Résumé

**Contexte:**

Cette étude visait à déterminer les facteurs associés aux piètres résultats thérapeutiques et à la fréquence des arythmies chez les patients atteints d'un syndrome hétérotaxique (SH).

**Méthodologie:**

Une étude de cohorte rétrospective menée dans un seul établissement de soins tertiaires a porté sur des patients ayant subi une intervention chirurgicale en vue de corriger un SH entre 2011 et 2020. Au total, 52 patients ont été admis dans l'étude. Les facteurs pertinents associés à la mortalité qui ont été retenus comprenaient le type morphologique (univentriculaire [UV] ou biventriculaire [BV]), la prématurité, le faible poids par rapport à l'âge au moment de l'intervention chirurgicale, une régurgitation valvulaire auriculoventriculaire, un retour veineux pulmonaire anormal et le type d'isomérisme auriculaire.

**Résultats:**

Trente-trois patients (63,4 %) ont reçu un diagnostic d'isomérisme auriculaire gauche (IAG), alors que les 19 patients restants (36,5 %) ont reçu un diagnostic d'isomérisme auriculaire droit (IAD). Trente-huit patients (73 %) ont subi une réparation UV, alors qu'une réparation BV a été réalisée chez 14 patients (27 %). Les patients ont fait l'objet d'un suivi d'une durée moyenne de 4,8 ans. En tout, 14 patients (27 %) sont décédés au cours de la période de suivi. Il est à noter que la plupart des patients ayant subi une réparation complète appartenaient au groupe IAG et que la survenue d'un bloc cardiaque de degré élevé a uniquement concerné les patients présentant un IAG. Qui plus est, ces patients étaient plus susceptibles de subir une réparation BV que les patients présentant un IAD, chez qui une réparation UV était plus fréquente (p = 0,008). Les anomalies veineuses pulmonaires sont en outre survenues à une fréquence plus élevée dans le groupe IAD. De façon prévisible, la présence d'un rythme non sinusal a été signalée fréquemment au sein de la cohorte. Finalement, le taux de mortalité était significativement plus élevé dans le groupe IAD (42 % vs 18 %, *p* = 0,06).

**Conclusion:**

La prise en charge des patients présentant un SH et une morphologie UV représente toujours un défi. Les facteurs de risque de mortalité comprenaient un âge précoce lors de la première intervention chirurgicale, et la présence d'un IAD (facteur presque significatif). Ces données pourraient faciliter la stratification du risque et les conseils aux patients.

Heterotaxy syndrome (HS) is defined as an abnormal arrangement of viscera across the left-right axis that differs from situs solitus and situs inversus totalis.^1^, ^2^, ^3^, ^4^, ^5^, ^6^, ^7^, ^8^, ^9^, ^10^, ^11^ This rare condition, estimated to impact 1 to 5000-7000 live births, has 2 variants: right atrial isomerism (RAI) and left atrial isomerism (LAI).^1^^,^^4^, ^5^, ^6^^,^^11^ Both variants are associated with complex intracardiac abnormalities; however, RAI is linked to a higher incidence of univentricular (UV) physiology, complete atrioventricular (AV) septal defect, total anomalous pulmonary venous drainage (TAPVD), and pulmonary stenosis.^1^^,^^4^, ^5^, ^6^^,^^11^

This study aims to present the most recent experience in managing patients with HS, along with midterm outcomes at Chest Diseases Hospital in Kuwait, which is the only hospital to provide congenital cardiac surgeries in the country. Risk factors (RFs) for mortality were assessed, alongside documenting arrhythmia incidence throughout follow-up.

## Methods

### Study population

A retrospective cohort study in a single tertiary center was conducted and included patients with operated HS between 2011 and 2020 with follow-up until March 2024. Such patients were identified using a surgical database, created in 2008, which incorporated all pediatric cardiac surgeries performed at Chest Diseases Hospital. HS was further confirmed through review of digital patient files and imaging, with patients categorized as having either RAI and LAI. RAI is characterized by bilateral right-sided atrial appendages, trilobed lungs, a midline liver, and frequently, bilateral superior vena cava, whereas LAI features bilateral left-sided atrial appendages, bilobed lungs, interrupted inferior vena cava, and polysplenia.^12^

Inclusion criteria were patients with HS who underwent primary cardiovascular repair at our center during the study period. Exclusion criteria included all patients who underwent primary cardiovascular repair at an outside institution or patients with situs inversus totalis. Each subject was assigned a unique identifier number, which was used to reference the data during analysis. This study was approved by the Ministry of Health Standing Committee for Coordination of Health and Medical Research.

### Variables

Data collected for each patient included demographic information (gestational age, birth weight, and sex), clinical characteristics (age and weight at the time of initial surgery, type of atrial isomerism, presence of dextrocardia, and fetal diagnosis status), anatomic and echocardiographic findings (AV septal defect, pulmonary venous anatomy, presence, and degree of AV valve regurgitation), and perioperative variables (type of surgical procedure, cardiopulmonary bypass, and aortic cross-clamp times). Postoperative data, including duration of mechanical ventilatory support, in-hospital mortality, and other surgical complications, were recorded. Rhythm status (presence of nonsinus atrial rhythm, high-grade heart block (HB), and supraventricular tachycardia [SVT]) was also recorded. For some patients, computed tomography and/or cardiac catheterization were performed to better delineate the cardiac anatomy. Follow-up information was obtained from clinical notes and imaging during routine outpatient visits.

The primary outcome was all-cause mortality during follow-up. Secondary outcomes included the occurrence of significant arrhythmias and postoperative complications. Factors assessed for association with mortality included type of atrial isomerism, type of physiology (UV or biventricular [BV]), prematurity, low birth weight, early age or weight at surgery, presence of AV valve regurgitation, and anomalous pulmonary venous return.

### Statistical analysis

Statistical analysis was performed with the IBM Statistical Product and Service Solution 26 (SPSS; IBM Corporations, Armonk, NY). Continuous variables were expressed as median, with interquartile ranges [Q1-Q3] in brackets. Differences in categorical variables were compared using the χ^2^ or the Fisher exact test, and the Mann-Whitney *U* test, Kruskal-Wallis test, or analysis of variance test was used to compare continuous variables, as appropriate. Patient data were processed through Kaplan-Meier survival analysis. RFs for mortality were identified by using Cox proportional hazards regression. A *P* value of <0.05 was considered significant and was used for variable inclusion on multivariate analysis.

## Results

Fifty-two patients were included in the final data analysis. Thirty-three patients (63.4%) were diagnosed with LAI, whereas the remaining 19 (36.5%) were diagnosed with RAI. Table 1 illustrates the characteristics of the entire cohort. Very few patients had fetal diagnosis. The median age at diagnosis was 1 day (0-6.5 days). Thirty-eight patients (73%) underwent UV repair, whereas 14 patients (27%) had BV repair. Eight patients (15.2%) underwent a bidirectional Glenn procedure, and 17 patients (32.7%) eventually received Fontan palliation. The median age at the initial operation was 19 days (5-144 days), and the median operation weight was 3.7 kg (2.8-5 kg). Patients were followed up for an average of 4.8 years (0-12.8 years). Lastly, 14 patients (27%) had died during the follow-up period.

**Table 1:** Main preoperative characteristics and types of surgical palliation or complete repair for the entire cohort including intraoperative data, mortality, and follow-up per type of atrial isomerism

Characteristics	RAI (n = 19)	LAI (n = 33)	*P* value
Fetal diagnosis, n (%)	1 (5)	9 (27)	0.053
Age at diagnosis (median/d)	1	1	0.917
Initial operation age (median/d)	18	20	0.918
Initial operation weight (median/kg)	3.2	3.7	0.423
Gestational age (wk)	38	37	0.193
Pulmonary venous anomalies	7 (6 SV patients)	5 (all BV patients)	0.074
Obstructive TAPVD, n (%)	2 (11)	0	0.057
Initial surgery, n (%)			
BTS	10 (53)	10 (30)	0.111
PAB	1 (5)	6 (18)	0.189
Norwood	1 (5)	4 (12)	0.419
BDG	3 (16)	0	0.019
TAPVD repair	1 (5)	2 (6)	0.906
TAPVD repair/BTS	1 (5)	0	0.183
TAPVD/BDG	1 (5)	0	0.183
Hybrid	0	2 (6)	0.274
PM	0	2 (6)	0.274
PM/PAB	0	1 (3)	0.444
AVSD repair	0	1 (3)	0.444
POTTS shunt	1 (5)	0	0.183
PAPVD/VSD repair	0	2 (6)	0.274
DORV/PS (TAP)	0	1 (3)	0.444
Kawashima	0	1 (3)	0.444
VSD closure	0	1 (3)	0.444
Biventricular repair, n (%)	1 (5)	13 (39)	0.008
Univentricular repair, n (%)	18 (95)	20 (61)	0.008
Bidirectional Glenn, n (%)	3 (16)	5 (15)	0.951
Fontan, n (%)	10 (53)	7 (21)	0.02
CPB/ACC time (min)	66.5/31	78/51	0.462/0.062
Nonsinus atrial rhythm, n (%)	10 (53)	17 (52)	0.938
SVT, n (%)	3 (16)	5 (15)	0.956
2:1 or higher grade HB, n (%)	0	4 (12)	0.114
Dextrocardia, n (%)	11 (58)	11 (33)	0.084
Death, n (%)	8 (42)	6 (18)	0.061
Follow-up (y)	4	5.3	0.258

ACC, aortic cross-clamp; AVSD, atrioventricular septal defect; BDG, bidirectional Glenn; BTS, Blalock-Taussig shunt; BV, biventricular; CPB, cardiopulmonary bypass; DORV, double outlet right ventricle; HB, heart block; LAI, left atrial isomerism; PAB, pulmonary artery banding; PAPVD, partial anomalous pulmonary venous drainage; PM, pacemaker; PS, pulmonary valve stenosis; RAI, right atrial isomerism; SV, single ventricle; SVT, supraventricular tachycardia; TAP, transannular patch; TAPVD, total anomalous pulmonary venous drainage; VSD, ventricular septal defect.

### Surgical results

Surgical and intraoperative data are summarized in Table 1. Notably, most patients with complete repair are among the LAI group, and high-grade HB only occurred in patients with LAI. Moreover, patients with LAI were more likely to receive a BV repair (13/33 [39%]), compared with patients with RAI (1/19 [5%]), whereas patients with RAI were more likely to receive UV repair (18/19 [95%]), compared with patients with LAI (20/33 [61%]) (*P* = 0.008). In addition, pulmonary venous anomalies occurred more frequently among the RAI vs the LAI groups (37% vs 15%, *P* = 0.074). Furthermore, the malignant combination of obstructed anomalous pulmonary venous return with single-ventricle (SV) physiology only occurred in the RAI cohort (11%, *P* = 0.057). As expected, nonsinus rhythm was frequently present among the cohort (approximately 50%). In addition, dextrocardia was more associated with RAI (58% vs 33%, *P* = 0.084). Finally, the mortality rate was significantly higher among the RAI group than the LAI cohort (42% vs 18%, *P* = 0.06).

Table 2 shows the postoperative complications identified from the patient data after undergoing an initial surgical repair. Eight different complications were identified, and their rates are shown for the total patient group and the subgroups. The most common identified complication was the need for prolonged mechanical ventilatory support (>72 hours), which was identified in 34.6% of the total patient group (*P* = 0.8). The small sample size of our cohort limited the statistical significance of the complication rates with the exception of “intraventricular hemorrhage/clot,” which was identified in 15.8% of patients with RAI compared with none among the LAI cohort (*P* = 0.02).

**Table 2:** Initial postoperative data and mortality per type of atrial isomerism

Initial repair	Total (n = 52)	RAI (n = 19)	LAI (n = 33)	*P* value
*Postoperative data*				
Complications, n (%)				
Sternum left open	6 (11.5)	3 (15.8)	3 (9.1)	0.46
Hypoxia	8 (15.4)	3 (15.8)	5 (15.2)	0.96
Prolonged mechanical ventilatory support (>72 h)	18 (34.6)	7 (36.8)	11 (33.3)	0.80
Wound infection	4 (7.8)	0 (0)	4 (12.1)	0.11
Pleural effusion	11 (21.2)	4 (21.1)	7 (21.2)	>0.99
Diaphragmatic palsy	4 (7.7)	1 (5.3)	3 (9.1)	0.25
Bleeding	11 (21.2)	5 (26.3)	6 (18.2)	0.48
IVH/clot	3 (5.8)	3 (15.8)	0 (0)	0.02

IVH, intraventricular hemorrhage; LAI, left atrial isomerism; n, number; RAI, right atrial isomerism.

### Tachy/bradyarrhythmia

At least 27 patients had documented nonsinus atrial rhythm during their follow-up (equally split between both groups), representing around 50% of the cohort. In terms of tachyarrhythmia, 8 patients had documented sustained SVT that required medical management during their follow-up (approximately 15% of each group), and 1 patient had evidence of twin AV nodes. On the other hand, 2 patients had postoperative HB, and another patient had congenital HB requiring pacemaker insertion; otherwise, 1 patient had 2:1 conduction postoperatively without the need for permanent pacing. As previously mentioned, all patients with bradyarrhythmia had LAI-type HS.

### Pulmonary venous anomalies

Twelve patients (23%) had anomalous pulmonary venous anomalies (Table 1): supracardiac (nonobstructive) in 3, intracardiac (nonobstructive) in 7, and infracardiac (obstructive) in 2. TAPVD was present in 9 patients, and the remaining 3 patients had partial anomalous pulmonary venous return. All patients with partial anomalous pulmonary venous return were associated with LAI, and all patients with LAI-TAPVD combined had BV physiology.

### Mortality

Table 3 describes all the deceased patient anatomies, palliative or corrective procedures performed, morbidities, timing, and cause of death. All deceased patients were palliated, and none had complete repair. All but one died while being admitted following a specific procedure.

**Table 3:** Diagnosis, surgical procedures, morbidities, and causes of death

subject	Diagnosis	Operation	Major morbidity	mortality	Cause of death
1	RAI, unbalanced AVSD, TAPVD to LSVC, b/l SVC, PS, malposed GA	BTS → vertical vein stent → TAPVD repair, BDG (left), ligation of RSVC and MPA → Redo TAPVD repair → fenestrated Fontan, AVV annuloplasty	Dextrocardia, brain infarction, convulsions, tracheostomy, cardiac arrest, open chest	Hospital death (immediate postoperative)	Desaturation, hypotension, VF
2	LAI, HLHS, interrupted IVC	Norwood/BTS	Severe bleeding	Hospital death (immediate postoperative)	Mediastinitis, sepsis
3	RAI, HLHS variant, AS, LSVC to common atrium, HAA	Norwood/BTS	Dextrocardia, high coronaries takeoff	Intraoperative death	Decreased CO, hypoxia
4	RAI, AVSD (balanced), PA, infracardiac obstructive TAPVD	TAPVD repair, BTS	Bleeding and cardiac arrest, open sternum	Hospital death	Hypoxia, sepsis
5	LAI, unbalanced AVSD, interrupted IVC, RAA with aberrant subclavian, congenital complete HB	Temporary PM → permanent PM, PA banding → PA band tightening → Kawashima procedure	Dextrocardia, sternal wound gapping/oozing. Pulmonary hypertensive crisis	Hospital death	Hypoxia
6	RAI, DORV, PS, AVSD, b/l SVC, interrupted IVC, TAPVD to RA	BDG → nonfenestrated Fontan → AVV repair	Moderate—severe AVVR, cardiac arrest	Hospital death	Fontan failure (generalized edema with poor function)
7	LAI, unbalanced AVSD, PS, b/l SVC	BTS → BDG	Dextrocardia, SVT	Hospital death	Died overseas
8	LAI, MAPCAS, VSD, PA, b/l SVC	BTS and PA plasty		Hospital death	Sepsis and multiorgan failure
9	RAI, unbalanced AVSD, PA	BTS	35 weeks GA	Hospital death	Shunt obstruction
10	RAI, AVSD, PA, hypoplastic branch PAs, MAPCAS, b/l SVC, RAA	POTTS shunt	Dextrocardia	Late death	NA
11	RAI, unbalanced AVSD, PA	BTS		Hospital death	Aspiration pneumonia
12	RAI, unbalanced AVSD	PA banding → BDG → nonfenestrated Fontan → Fontan + RPA stenting	Dextrocardia, protein losing enteropathy	Hospital death	Depressed function, cardiac arrest
13	LAI, unbalanced AVSD, PA, b/l SVC	BTS	SVT	Hospital death	Sepsis, pneumothorax, hypoxia, bradycardia, hypotension, acidosis
14	LAI, CAVB, partial AVSD	PM	Anasarca, candidemia	Hospital death	Sepsis, heart failure

AS, aortic stenosis; AVSD, atrioventricular septal defect; AVV, atrioventricular valve; AVVR, AVV regurgitation; b/l SVC, bilateral superior vena cava; BDG, bidirectional Glenn; BTS, Blalock-Taussig shunt; CAVB, complete atrioventricular block; CO, cardiac output; DORV, double outlet right ventricle; GA, great arteries; HAA, hypoplastic aortic arch; HB, heart block; HLHS, hypoplastic left heart syndrome; IVC, inferior vena cava; LAI, left atrial isomerism; LSVC, left superior vena cava; MAPCAS, major aortopulmonary collateral arteries; MPA, main pulmonary artery; NA, not available; PA, pulmonary atresia; PM, pacemaker; PS, pulmonary valve stenosis; RAA, right aortic arch; RAI, right atrial isomerism; RPA, right pulmonary artery; RSVC, right superior vena cava; SVT, supraventricular tachycardia; TAPVD, total anomalous pulmonary venous drainage; VF, ventricular fibrillation; VSD, ventricular septal defect.

**Figure 1:**
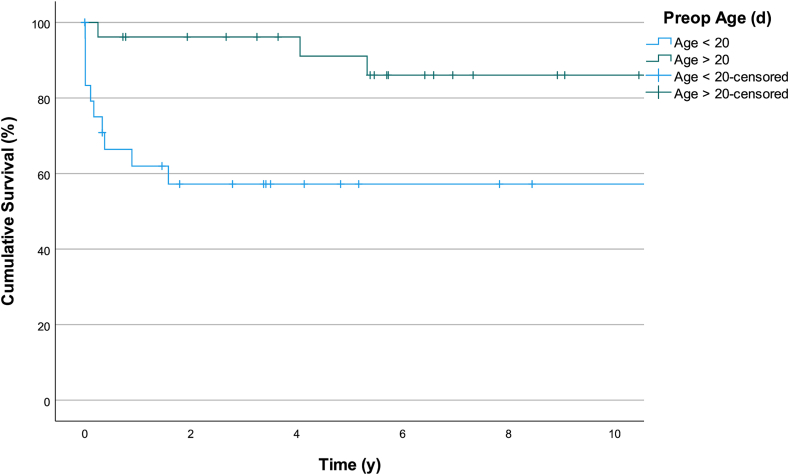
Kaplan-Meier curve survival analysis for patients undergoing surgery before 20 days of age vs beyond 20 days of age.

**Figure 2:**
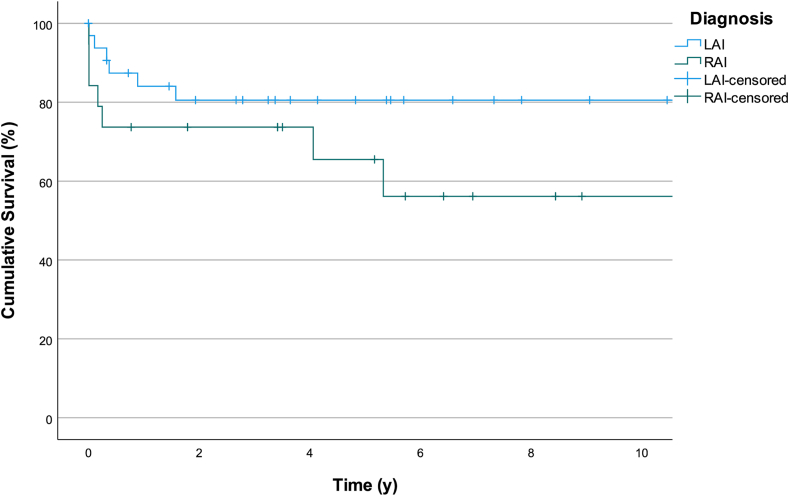
Kaplan-Meier curve survival analysis for patients with RAI vs LAI. LAI, left atrial isomerism; RAI, right atrial isomerism.

**Figure 3:**
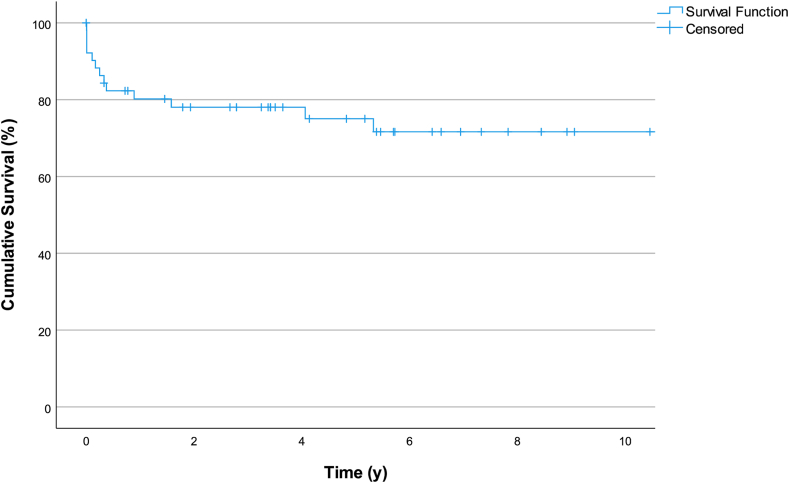
Kaplan-Meier curve survival analysis for the entire cohort.

Relevant factors associated with mortality that were considered included UV or BV physiology, prematurity, low weight/age at surgery, presence of AV valve regurgitation, anomalous pulmonary veins drainage, and type of atrial isomerism. Table 4 demonstrates univariate survival analysis of the collected patient data with HR of 4.12 for patients with operated age < 20 days compared to operated age > 20 days (*P* = 0.019) [Fig. 1]. Figure 2 and Figure 3 shows survival rates for RAI vs LAI and survival analysis for the entire cohort.

**Table 4:** Factors associated with mortality

Outcome	Univariate analysis		
	HR	[95% CI]	*P*
Operation weight (kg)			
<2.5	0.59	[0.75-4.59]	0.612
>2.5	1.00	[Reference]	
Operation age (d)			
<20	4.12	[1.26-13.41]	0.019
>20	1.00	[Reference]	
Gestational age			
Preterm	0.77	[0.16-3.62]	0.740
Full term	1.00	[Reference]	
Birth (kg) Weight			
>2.5	3.486	[0.436-27.873]	0.239
<2.5	1.00	[Reference]	
Diagnosis			
RAI	2.758	[0.946-8.043]	0.063
LAI	1.00	[Reference]	
Procedure			
SV	1.43	[0.40-5.14]	0.581
BV	1.00	[Reference]	
AVVR			
Significant	1.076	[0.24-4.86]	0.92
Nonsignificant	1.00	[Reference]	
TAPVD			
Present	0.87	[0.19-3.91]	0.85
Absent	1.00	[Reference]	

AVVR, atrioventricular valve regurgitation; BV, biventricular; CI, confidence interval; HR, hazard ratio; LAI, left atrial isomerism; RAI, right atrial isomerism; SV, single ventricle; TAPVD, total anomalous pulmonary venous drainage.

## Discussion

The incidence for HS in the country was similar to previous publications with a reported incidence of around 1%-3% among patients with congenital heart disease.^1^^,^^5^ Specifically, LAI was more prevalent than RAI, similar to other reports from the United States and Europe, but unlike reports from East Asia.^8^ Herein, we describe a single center’s most recent experience, aiming to review the surgical results, midterm outcomes, and assess RFs for mortality in patients with HS.

The main observation within this cohort is the high mortality rate. Nevertheless, published mortality rates in the literature may reach up to 40% depending on duration of follow-up, surgical era, institutional expertise, and complexity of the cohort.^1^, ^2^, ^3^, ^4^, ^5^^,^^11^^,^^13^ However, none of the mortalities occurred among the BV group, suggesting that mortality is primarily due to the complex subset of SV patients with HS. Undoubtedly, HS is frequently associated with UV physiology.^1^^,^^3^, ^4^, ^5^, ^6^^,^^11^^,^^13^ Furthermore, there is a high rate of associated pulmonary venous anomalies complicating patients’ surgical procedures.^3^^,^^5^^,^^13^ Specifically, to this cohort, the low rate of fetal diagnosis, birth at noncardiac centers, care provided at tertiary cardiac center and not multispecialty children’s hospital, and the unavailability of cardiac transplant option might have contributed to the increased mortality. RAI was associated with greater mortality due to the highly associated SV physiology (95%) and the higher incidence of associated pulmonary venous anomalies (37%), 2 of which had the malignant combination of obstruction and UV physiology.^11^^,^^14^ Similarly, globally the mortality of HS patients with congenital heart disease remains high, despite improvement in the current era.^1^^,^^2^^,^^6^ This improvement is likely due to the advances in the diagnostic tools, cardiac surgical techniques, and the intensive care milieu.^2^

The highest complication rate was prolonged intubation times. Understandably, this could be partly related to the associated ciliary dyskinesia in patients with HS^2^^,^^4^^,^^5^^,^^11^^,^^13^ and the accompanying reactive pulmonary vasculature, in addition to the intrinsically smaller pulmonary veins at least among the patients with RAI.^6^^,^^11^ Moreover, the frequent association with anomalous pulmonary venous anomalies (23%) in patients with HS may also prolong the intubation times.^1^^,^^3^^,^^4^^,^^6^^,^^11^^,^^13^

Independent RFs included lower operation age (Table 4), which is similar to other reports.^2^, ^3^, ^4^, ^5^ Other RFs analyzed did not reach statistical significance, likely secondary to the low number of studied patients. Specifically, the type of atrial isomerism, specifically RAI, appeared to approach significance on univariate analysis. Indeed, RAI is a surrogate for more complex cardiac anatomies (Table 2) with UV physiology and hence is associated with poorer prognosis.^1^^,^^2^^,^^6^

HB and LAI association is further seen in this cohort, especially when associated with abnormal AV septation.^7^^,^^8^^,^^15^, ^16^, ^17^, ^18^ Indeed, 3 of the 4 patients with HB in this cohort had abnormal septation. None of the RAI had HB similar to other report,^15^ perhaps related to the more frequent incidence of twin AV nodes among patients with RAI.^8^^,^^15^ An absent sinus node frequently seen in patients with LAI and bilateral sinus nodes commonly seen in patients with RAI explain the high incidence of non-right sinus rhythm observed in this cohort.^8^, ^9^, ^10^^,^^18^^,^^19^

Finally, patients frequently developed SVT during the study period, mostly among the subset of UV patients, similar to the largest report available^20^ but unlike other reports.^7^ It is well known that patients with HS, especially RAI type, have twin AV nodes and, hence, are predisposed to nodal to nodal reentry tachycardia.^7^^,^^8^^,^^15^^,^^18^^,^^20^^,^^21^

### Limitations

Limitations of this study included the small sample size. The small number of subjects limits statistical power and the feasibility of subgroup analysis in the cohort of the current study. The retrospective nature of the study limits the study with the risk for selection bias. Causation assessment was not possible. The usual limitations of a single-center retrospective study, such as interobserver variability for clinical and echocardiographic evaluations performed by different physicians, apply to this data analysis.

## Conclusion

Managing HS patients with UV physiology still remains a challenge in this day and age. RFs for mortality included RAI and lower initial operation age. Postoperatively, prolonged intubation times were the commonest complications observed. These data may assist with risk stratification and patient counseling.
